# Epiphytic and endophytic bacteria on *Camellia oleifera* phyllosphere: exploring region and cultivar effect

**DOI:** 10.1186/s12862-024-02240-3

**Published:** 2024-05-13

**Authors:** Xiaolin Chen, Lili Li, Yuanhao He

**Affiliations:** 1 Key Laboratory of National Forestry and Grassland Administration On Control of Artificial Forest Diseases and Pests in South China, Hunan Provincial Key Laboratory for Control of Forest Diseases and Pests, Key Laboratory for Non-Wood Forest Cultivation and Conservation of Ministry of Education, Key Laboratory of Forest Bio-Resources and Integrated Pest Management for Higher Education in Hunan Province, College of Forestry, Central South University of Forestry and Technology, Changsha, China; 2 Ordos Forestry and Grassland Development Center, Ordos, China

**Keywords:** *Camellia oleifera*, Phyllosphere, Endosphere, Microbial community, Plant–microbe interactions

## Abstract

The epiphytic and endophytic bacteria play an important role in the healthy growth of plants. Both plant species and growth environmental influence the bacterial population diversity, yet it is inconclusive whether it is the former or the latter that has a greater impact. To explore the communities of the epiphytic and endophytic microbes in *Camellia oleifera*, this study assessed three representative *C. oleifera* cultivars from three areas in Hunan, China by Illumina high-throughput sequencing. The results showed that the diversity and species richness of endophytic microbial community in leaves were significantly higher than those of microbial community in the epiphytic. The diversity and species richness of epiphytic and endophytic microbes are complex when the same cultivar was grown in different areas. The *C. oleifera* cultivars grown in Youxian had the highest diversity of epiphytic microbial community, but the lowest abundance, while the cultivars grown in Changsha had the highest diversity and species richness of endophytic microbes in leaves. It was concluded that the dominant phylum mainly included Proteobacteria, Actinobacteriota and Firmicutes through the analysis of the epiphytic and endophytic microbial communities of *C. oleifera*. The species and relative abundances of epiphytic and endophytic microbial community were extremely different at the genus level. The analysis of NMDS map and PERMANOVA shows that the species richness and diversity of microbial communities in epiphytes are greatly influenced by region. However, the community structure of endophytic microorganisms in leaves is influenced by region and cultivated varieties, but the influence of cultivars is more significant. Molecular ecological network analysis showed that the symbiotic interaction of epiphytic microbial community was more complex.

**Supplementary Information:**

The online version contains supplementary material available at 10.1186/s12862-024-02240-3.

## Introduction

Plants are densely colonized by a variety of microbes [1], some of which the epiphytes stay on the surface of plant organs, while others are able to penetrate further inside the plants and are called endophytes [2]. Compared with the rhizosphere environment, the phyllosphere environment is relatively deficient in nutrients and water resources, and the phyllosphere microorganisms face harsh conditions such as ultraviolet radiation, excessive temperature difference and reactive oxygen species, which are not conducive to their growth. Even so, the composition of phyllosphere microbial communities is still rich and complex, particularly, the diversity of microbial communities differs within different species [3, 4]. Interactions between phyllosphere microbes have a critical impact on plant growth and crop yield [5].

Endophytes refer to fungi or bacteria that live in various tissues of plants at a certain stage or all stages of the life cycle without causing obvious disease symptoms of the host, and establish a symbiotic relationship with the plants [6]. Endophytes can promote plant growth, induce disease resistance, improve the quality of agricultural products, play a role in biological nitrogen fixation and reduce harmful compounds. It is an important source of biological control agents [7, 8].

The structure and diversity of epiphytes and endophytic microbial communities are mainly affected by plant species and environmental factors. Accroding to Laforest-Lapointe et al. [9], there was a significant correlation between the phyllosphere microbial community and plant phenotype. Related studies on the differences in the composition of epiphytes communities between different genotypes of the same species have mainly focused on forests or horticultural trees in temperate regions, and the results have been conflicting. For example, Hunter et al. [10] detected differences in leaf bacterial community composition between lettuce cultivars, while Rastogi et al. [11] did not obtain the same results. Similarly, endophytic bacterial community structures vary widely across plant genotypes. As an example, studies by Hardoim et al. [12] showed that genotype largely determined the composition of endophytic bacterial communities in different rice cultivars. The study by Lamit et al. [13] also showed that the genotype of narrow-leaved *Populus* affected its shoot endophytic fungi abundance and community composition. The phyllophyte microbes face nutrient deficiencies and variable environmental conditions, primarily the temperature, humidity, and radiation that are constantly changing. Herrmann et al. [14] found that both position in the canopy and tree species have a strong effect on the structure of epiphytes communities in a floodplain hardwood forest, that is, consistently lower bacterial diversity at the top of the canopy compared to the canopy mid. It is worth mentioning that endophytic microorganisms, compared with epiphytes microorganisms, live under more stable conditions, but their community composition also differs to a certain extent. Taking the study of Xu [15] as an example, the endophytes of *Stipa* from six grassland plots were isolated and cultured, and the results showed that their endophytic bacterial community structures were absolutely different. However, it remains to be elucidated which factors have a greater impact on the epiphytes and the leaf endophytic microbial community structures by plant species and environmental characteristics.

*C. oleifera* is an evergreen woody edible oil tree species that is widely grown in the subtropical regions of China [16]. The tea oil extracted from *C. oleifera* is commonly used as vegetable oil in southern China. There are various types of pests and diseases of *C. oleifera*. At present, there are 42 well-known *Camellia* diseases, 35 of which occur on leaves, such as anthracnose and soft rot. The different community structures of epiphytic and endophytic microorganisms are closely related to host diseases. For example, Chen et al. [17] found that the damaged genetic network of *Arabidopsis thaliana* changed the composition and diversity of the epiphytic microbial community, and the imbalance of the microbial community led to yellowing and necrosis of leaves. Zhou et al. [18] also confirmed that the bacterial diversity in the phyllosphere of healthy *Eupatorium adenophorum* was higher than that of diseased plants, and the fungal and bacterial community structures in the phyllosphere of healthy and diseased plants were different. Similar results were presented in the study of Gao et al. [19], the diversity of endophytic fungi and bacteria in the stalks of sugarcane cultivars resistant to ratoon stunting disease was rich, and the community composition was especially different. Therefore, it is necessary to investigate the influence of *C. oleifera* cultivars and environmental characteristics on the diversity and community structures of epiphytic and endophytic microorganisms. At present, the research on the epiphytic microbes of *C. oleifera* is relatively blank. The research on endophytes in *C. oleifera* mainly focuses on the community changes after being infected by pathogens, but it is not related to plant species and environmental factors. Cui et al. [20] found that the occurrence of *C. oleifera* anthracnose changed the community structure of endophytic bacteria in *C. oleifer*a leaves, allowing a few disease-resistant related species to grow dominantly.

In this study, the *C. oleifera* cultivars grown in the subtropical region of southern China were used as the research objects, and the community structures of epiphytes and leaf endophytic microorganisms was analyzed through high-throughput sequencing technology. The objectives of this study include, (1) What are the differences in the diversity and composition of epiphytic and endophytic microbial communities among different *C. oleifera* cultivars? (2) What are the differences in the diversity and composition of microbial communities within the same *C. oleifera* cultivars in different region? (3) Are the epiphytic community and the endophytic community mainly affected by region or plant cultivars? We revealed potential key factors affecting the microbial communities of *C. oleifera* leaves, and anticipated possible applications of these microbial communities in future interaction studies.

## Materials and methods

### Sample collection

Three *C. oleifera* cultivars, namely 'Youxian' 'Huashuo', and 'Xianglin 210', were collected from Youxian (N 41° 32.593′, W 07° 07.445′, A), Wangcheng (N 41° 32.756′, W 07° 07.590′, B), and Changsha (N 41° 29.454′, W07° 30.398′, C), in Hunan Province, China with similar management measures and similar altitudes in May, June, and July 2020. For sample collection: apparently, the mature healthy fourth and fifth leaves from the apical leaf were randomly collected with sterilized shears and gloves, placed into sterile roll bags and brought to the lab on ice. Plant material was stored at –20 °C refrigerator for subsequent analysis. There were 54 samples in total, 27 epiphytic samples and 27 endophytic samples, each including three replicates. The appraiser of *C. oleifera* region was Professor Zou Feng of Central South University of Forestry and Technology. Plant samples were not kept in a publicly available herbarium.

For epiphytic microorganisms: weighed 10 g of the leaf samples, cut them into pieces and put into a sterile conical flask, then added 100 ml of 0.1 M potassium phosphate buffer (pH = 8.0). The samples were ultrasonically washed for 1 min, vortexed for 10 s, and this step was repeated twice. Took out the washed samples, and then repeated the above steps one more time [21]. The washed samples were mixed and filtered through 0.22 μm filter membrane. The filtered membrane was quick-frozen with liquid nitrogen and stored at − 80 °C. The plant samples were washed twice with 70% ethanol and stored at − 80 °C for experiments related with epiphytic microorganisms.

For leaf endophytic microorganisms: weighed 10 g of the leaf samples, washed the samples with sterile water for 30 s, then soaked them in 70% ethanol solution for 2 min, and in 2.5% NaClO (containing 0.1% Tween80) for 5 min, transfered them to 70% sterile ethanol for 30 s, and then washed plant tissues 3 times with sterile water. Nucleic acid extraction and quick freezing in liquid nitrogen were performed and samples were stored at -80 °C for experiments related with leaf endophytic microorganisms.

### DNA extraction and PCR amplification

Microbial DNA was extracted from 250 mg of endophytic leaf and filter membrane with epiphytic microorganisms using the Power Soil DNA Isolation Kit, following the protocol provided by the manufacturer (MoBio, Carlsbad, CA, United States). The final DNA concentration and purity were assessed using a NanoDrop 2000 ultra violet-visual (UVvis) spectrophotometer (Thermo Scientific, Wilmington, DE, United States), and the DNA quality was checked using 1% agarose gel electrophoresis. DNA integrity was verified by gel electrophoresis using 0.8% agarose gel. Primers 515F (5'-GTGCCAGCMGCCGCGGTAA-3'), and 907R (5'-CCGTCAATTCMTTTRAGTTT-3') were used to amplify the 16S rRNA gene [22]. Each 25 μl PCR reaction contains 10 ng DNA, 250 μM dNTPs, 200 nM forward primer, 200 nM reverse primer, 12.5 μg Ambion Ultrapure BSA, FastPfu Buffer, and 1 U of TransStart FastPfu DNA Polymerase (TransGen). Cycling conditions were 94 °C for 3 min, followed by 25 cycles of 94 °C for 30 s, 55 °C for 30 s, and 72 °C for 45 s, with a final extension period of 10 min at 72 °C. All samples were amplified in triplicate. The PCR product was extracted from 2% agarose gel and purified using the AxyPrep DNA Gel Extraction Kit (Axygen Biosciences, Union City, CA, USA) according to manufacturer’s instructions and quantified using Quantus™ Fluorometer (Promega, USA).

### Illumina MiSeq sequencing

Purified amplicons were pooled in equimolar amounts and paired-end sequenced on an Illumina MiSeq PE300 platform (Illumina, San Diego,USA) according to the standard protocols by Majorbio Bio-Pharm Technology Co. Ltd. (Shanghai, China) [23–25].

### Data processing

Raw FASTQ files were de-multiplexed using an in-house perl script, and then quality-filtered by fastp version 0.19.6 [26] and merged by FLASH version 1.2.7 [22] with the following criteria:

(i) the 300 bp reads were truncated at any site receiving an average quality score of < 20 over a 50 bp sliding window, and the truncated reads shorter than 50 bp were discarded, reads containing ambiguous characters were also discarded; (ii) only overlapping sequences longer than 10 bp were assembled according to their overlapped sequence. The maximum mismatch ratio of overlap region is 0.2. Reads that could not be assembled were discarded. Then the optimized sequences were clustered into operational taxonomic units (OTUs) using UPARSE 7.1 [27, 28] with 97% sequence similarity level. The most abundant sequence for each OTU was selected as a representative sequence. The OTU table was manually filtered, i.e., chloroplast sequences in all samples were removed. To minimize the effects of sequencing depth on alpha and beta diversity measure, the number of 16 s rRNA gene sequences in each sample is small to the minimum sequencing depth. The taxonomy of each OTU representative sequence was analyzed by RDP Classifier version 2.2 [29] against the 16S rRNA gene database (Silva v138) using confidence threshold of 0.7.

### Statistical analysis

The analysis of the *C. oleifera* leaf endophytic and epiphytes bacterial communities was as follows. Significant differences in the variance of parameters were evaluated with ANOVA and Student’s t-test in SPSS 17.0. Post hoc comparisons were conducted by the Tukey’s honest significant differences tests. Student’s t-test was used to test the effect of epiphytic and endophytic bacterial diversity in each oleifera tree, *C. oleifera* cultivars (‘Huashuo’, ‘Xianglin210’, ‘Youxian’) and plant location (Changsha, Youxian, Wangcheng) on the read abundances. Bacteria with relative abundance < 0.01% of the sample was classified as other categories, and bacteria with relative abundance > 5.00% was referred to as the dominant bacteria. Based on the OTUs information, alpha diversity indices including observed Chao1 richness, Shannon index and Simpson index were calculated with Mothur v1.30.1 [30]. In Alpha diversity analysis, Chao index refers to community richness, Shannon and Simpson indices refer to community diversity. The higher the Shannon index value and the lower the Simpson index value, the higher the community diversity. Both NMDS and ANOSIM analyses were performed by using the Community Analysis Package v. 4.0 [31]. As a rule of thumb, a stress value below 0.2 is deemed good and reliable. The larger the R value, the more obvious the difference. Permutational multivariate analysis of variance (PERMANOVA) was used to test the effects of cultivar and or plant location on the epiphytic and endophytic microbiomes associated with using the adonis2 function in R [32]. The relative abundance of bacterial families that exhibited a significant (*p* < 0.05) differential abundance across host cultivar or plant location were represented in a heatmap using the heatmap.2 function in the gplots package of R software. Networkx software to analyze and construct networks between microorganisms. The PICRUSt function prediction was used to predict the functional composition of epiphytes and endophytic bacteria. The greengene id corresponding to each OTU, the COG, and KEGG functions of the OUT were annotated to obtain the function level of COG and KEGG, and the abundance information for each function in different samples.

## Results and analysis

### Sequencing data analysis

The bacterial community diversity of 27 epiphytes samples was analyzed by 16S rDNA high-throughput sequencing (each treatment had 3 replicates), and a total of 1,316,491 effective tags were obtained, with an average of 48,758 sequences per sample, including 31 phyla, 63 classes, 139 orders, 259 families, 451 genera, 671 species and 915 OTUs. The bacterial community diversity of 27 endophytic samples was analyzed by 16S rDNA high-throughput sequencing (each treatment had 3 replicates), and a total of 1,600,915 effective tags were obtained, with an average of 59,293 sequences per sample, including 37 phyla, 66 classes, 156 orders, 345 families, 720 genera, 1192 species and 1793 OTUs. The number of OTU of leaf endophytic bacteria was more than that of epiphytic microbes. When the sequencing depth was 30 000, the rarefaction curve of each sample tends to be saturated, indicating that new species were not going to be continuously being detected in the sample with increasing sequencing data (Supplement Fig. 1).

**Fig. 1:**
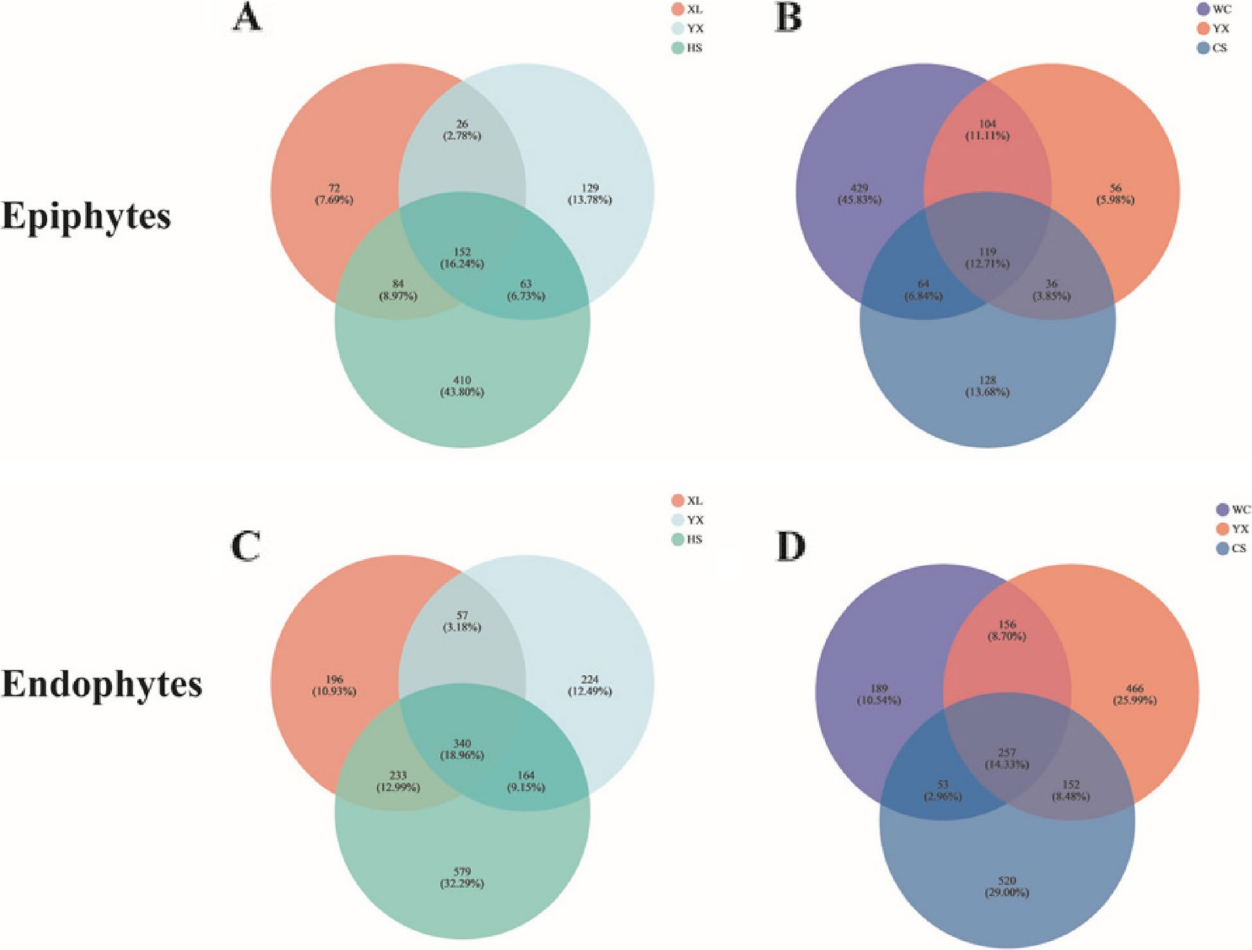
Venn diagram of OTUs of oil tea bacteria in different cultivars and regions. XL: Xianglin210 HS: Huashuo YX: Youxian CS: Changsha WC: Wangcheng YX: Youxian. **A** the epiphytic community of the same regions with different cultivars, **B** the epiphytic community of the same cultivars in different regions, **C** the endophytes community of the same regions with different cultivars, **D** the endophytes community of the same cultivars in different regions

### The abundance and diversity of epiphytic and endophytic microbial communities in *C. oleifera*

Alpha diversity index analysis was performed on epiphytic and endophytic microbial communities of *C. oleifera* based on operational taxonomic units (OTUs) classification. For the same region, comparisons were made between different cultivars (Table 1). In terms of epiphytes community, Shannon index and Simpson index results showed that the diversity of epiphytes bacterial community of 'Huashuo' was slightly higher than that of the other two cultivars. Chao index results showed that 'Huashuo' was the highest, with a significant difference from the other two cultivars, 2.11 times that of 'Youxian' cultivar, and 1.77 times that of 'Xianglin 210' cultivar. In general, the diversity and richness of the epiphytic community in 'Huashuo' were the highest, while that of the 'Youxian' was the lowest. In terms of leaf endophytic bacterial community, Shannon and Simpson index results showed that 'Huashuo' had the highest diversity and 'Xianglin 210' had the lowest. The Chao index results showed that 'Huashuo' was the highest, and 'Xianglin 210' was the lowest. The results suggest that, the leaf endophytic bacterial community of 'Huashuo' had the highest diversity and richness.

**Table 1:** Comparison of epiphytic and endophytic community between Xianglin210, Huashuo and Youxian cultivars regarding their Alpha diversity (Shannon’s index, Simpson’s indexand Chao’s index).Different lowercase letters in the same row indicate that there are significant differences in the epiphytic and endophytic between different varieties(*P* < 0.05)

Part	Varieties	Shannon index	Simpson index	Chao index
Epiphytes	YX	0.4958 ± 0.3661a	0.8150 ± 0.1305a	92.5031 ± 62.5391b
	XL	0.7132 ± 0.5023a	0.7378 ± 0.1754a	110.2332 ± 44.8418ab
	HS	0.7325 ± 0.5045a	0.7705 ± 0.1346a	195.9568 ± 130.9035a
Endophytes	YX	3.0533 ± 0.5650a	0.1416 ± 0.0499a	215.0914 ± 77.3337a
	XL	2.6394 ± 2.0683a	0.2582 ± 0.2429a	236.6079 ± 119.8757a
	HS	3.3557 ± 0.5117a	0.1076 ± 0.0485a	289.4447 ± 158.1115a

*XL* Xianglin210, *HS* Huashuo, *YX* Youxian, *CS* Changsha, *WC* Wangcheng, *YX* Youxian

Under the same cultivated variety, different areas were compared (Table 2). Compared with the Shannon index, the Shannon index in the leaf epiphytes community showed that Youxian area was the highest, and it was significantly different from Changsha area. Simpson index shows that Youxian is the lowest and Changsha is the highest, and there is a significant difference between Changsha and the other two regions. Chao index shows that Wangcheng is the highest and Youxian is the lowest. Therefore, the diversity of bacterial communities among the leaves is the highest in Youxian and the lowest in Changsha, while the richness of bacterial communities is the lowest in Youxian and the highest in Wangcheng. Comparing Shannon's index and Simpson's index, the diversity of bacterial community in Changsha area is slightly higher than that in other two areas. Chao index shows that Changsha is the highest, and there is a significant difference between Changsha and Wangcheng. Therefore, among the endophytic communities, the diversity and richness of bacteria in Changsha is the highest, that in Youxian is the lowest, and that in Wangcheng is the lowest.

**Table 2:** Comparison of epiphytic and endophytic community between Xianglin 210, Huashuo and Youxian cultivars regarding their Alpha diversity (Shannon’s index, Simpson’s index and Chao's index).Different lowercase letters in the same row indicate that there are significant differences in the epiphytic and endophytic between different region (*P* < 0.05)

Part	Varieties	Shannon index	Simpson index	Chao
Epiphytes	YX	0.8348 ± 0.1115a	0.6927 ± 0.1116b	126.9391 ± 69.4315a
	CS	0.6800 ± 0.1492b	0.8600 ± 0.1493a	115.4369 ± 84.0990a
	WC	0.6951 ± 0.1372ab	0.7705 ± 0.1372ab	156.3170 ± 477782a
Endophytes	YX	2.9138 ± 1.0693a	0.2122 ± 0.2093a	241.6333 ± 152.9947a
	CS	3.3308 ± 0.5854a	0.1117 ± 0.0659a	312.2632 ± 119.8618ab
	WC	2.8037 ± 0.5945a	0.18343 ± 0.1538a	187.2473 ± 43.0396b

*XL* Xianglin210, *HS* Huashuo, *YX* Youxian, *CS* Changsha, *WC* Wangcheng, *YX* Youxian

The results of Venn diagram analysis showed that in the same area, *C. oleifera* 'Huashuo' had the most unique OTUs (410 for epiphytes community and 579 for leaf endophytic bacterial community), and shared the most OTUs with the other two cultivars. 'Xianglin 210' cultivar had the fewest OTUs. ‘Youxian’ shared the least OTUs with the other two cultivars.

For the same cultivars, in terms of epiphytic community, the OTUs of Wangcheng samples were the most (429), and the OTUs of Youxian samples were the least (56). In terms of leaf endophytic bacterial community, the OTUs of Changsha samples were the most (520), and the OTUs unique to Wangcheng samples were the least (189) (Fig. 1).

Comprehensively considering Venn diagram and Alpha diversity analysis, the diversity and richness of epiphytic and leaf endophytic microbes of *C. oleifera* 'Huashuo' were higher than those of the other two cultivars, and the epiphytes and leaf endophytic community structures of *C. oleifera* in different regions were more complex.

### Analysis of the basic composition and structures of epiphytic and leaf endophytic microbial communities in *C. oleifera*

The relative abundances of epiphytic and leaf endophytic communities in *C. oleifera* were analyzed at the phylum and genus levels.

**Fig. 2:**
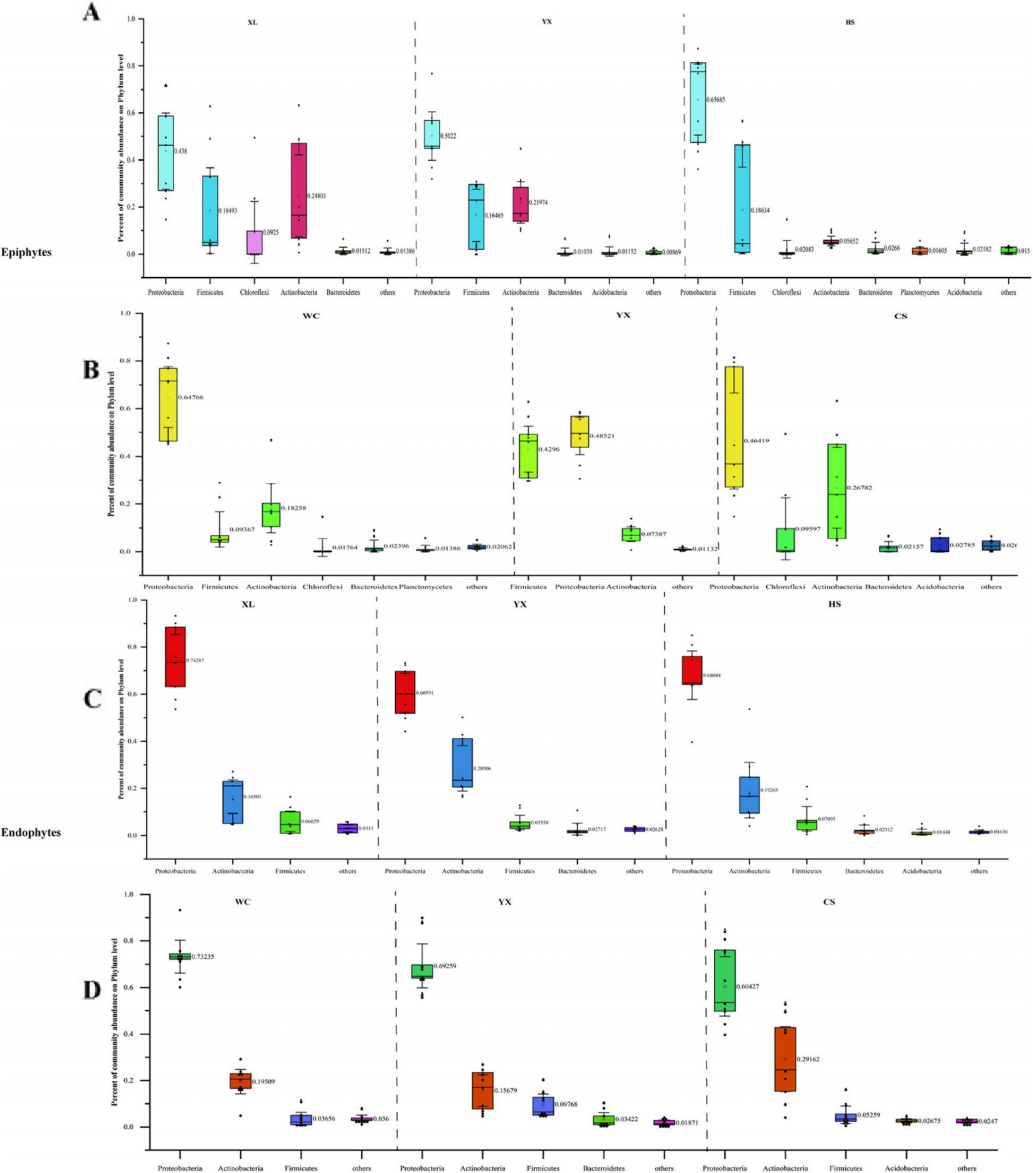
The epiphytic and endophytic bacterial phylum in different regions of *C. oleifera* are relatively abundant. XL: Xianglin210 HS: Huashuo YX: Youxian CS: Changsha WC: Wangcheng YX: Youxian. **A** the epiphytic community of the same regions with different cultivars, **B** the epiphytic community of the same cultivars in different regions, **C** the endophytes community of the same regions with different cultivars, **D** the endophytes community of the same cultivars in different regions

The predominant flora of the epiphytic community of these three *C. oleifera* cultivars included 4 phyla, namely Proteobacteria, Firmicutes, Chloroflexi and Actinobacteria, accounting for more than 80% of the total bacteria, but the proportion of different flora in each group of samples was slightly different. The relative abundance of Proteobacteria in epiphytic bacteria community is the highest, with the highest being 64.7% in Wangcheng area. (Fig. 2A, B). The relative abundance of Chloroflexi in the ‘Youxian’ cultivar was > 0.1%, which was lower than that of the other two cultivars; the relative abundance of Actinobacteria was 5.6% in the ‘Huashuo’ cultivar and 22.0% in the ‘Youxian’ cultivar (Fig. 2A).

The composition and relative abundance of epiphytic communities of *C. oleifera* planted in different regions were quite different. Firmicutes was dominant in samples from Youxian area, accounting for 43.0%, and respectively, < 1.0% in samples from Changsha area and 9.4% in samples from Wangcheng area. The relative abundance of Chloroflexi in samples from Changsha area was obviously high, accounting for 10% (Fig. 2B).

There were some differences in the composition of the endophytic and epiphytes communities. Firmicutes had a high relative abundance in the epiphytes bacterial community, and conversely had a low proportion in the leaf endophytic bacterial community. The dominant flora of the endophytic bacterial community mainly involved three phyla, namely, Proteobacteria, Actinobacteria and Firmicutes, accounting for more than 90% of the total bacteria, and the proportion of different flora in each group of samples was slightly different. The relative abundance of Proteobacteria was the highest, accounting for 74.3%, 60.6%, 68.0%, 73.2%, 69.3% and 60.4% of the samples, respectively (Fig. 2C, D). The dominant flora of different cultivars samples were basically the same, but the relative abundance was slightly different. For example, for ‘Xianglin 210’ samples, the relative abundance of Proteobacteria was high, while the relative abundance of Firmicutes was low. For ‘Youxian’ samples, the relative abundance of Actinobacteria was 28.5%, which was obviously higher than that of ‘Xianglin 210’ (16.5%) and ‘Huashuo’ (19.3%) (Fig. 2C).

The composition and relative abundance of endophytic bacterial communities of *C. oleifera* cultivars planted in different regions were different. The relative abundance of Actinobacteria (29.2%) in Changsha samples was higher than that of the other two areas, while the relative abundance of Bacterioidetes (> 1%) was the lowest (Fig. 2D).

At the phylum level, the dominant flora of epiphytes and leaf endophytic communities were different, mainly the Chloroflexi. Although the dominant flora of epiphytes bacterial communities from different areas were the same, the relative abundances were notably different.

**Fig.3:**
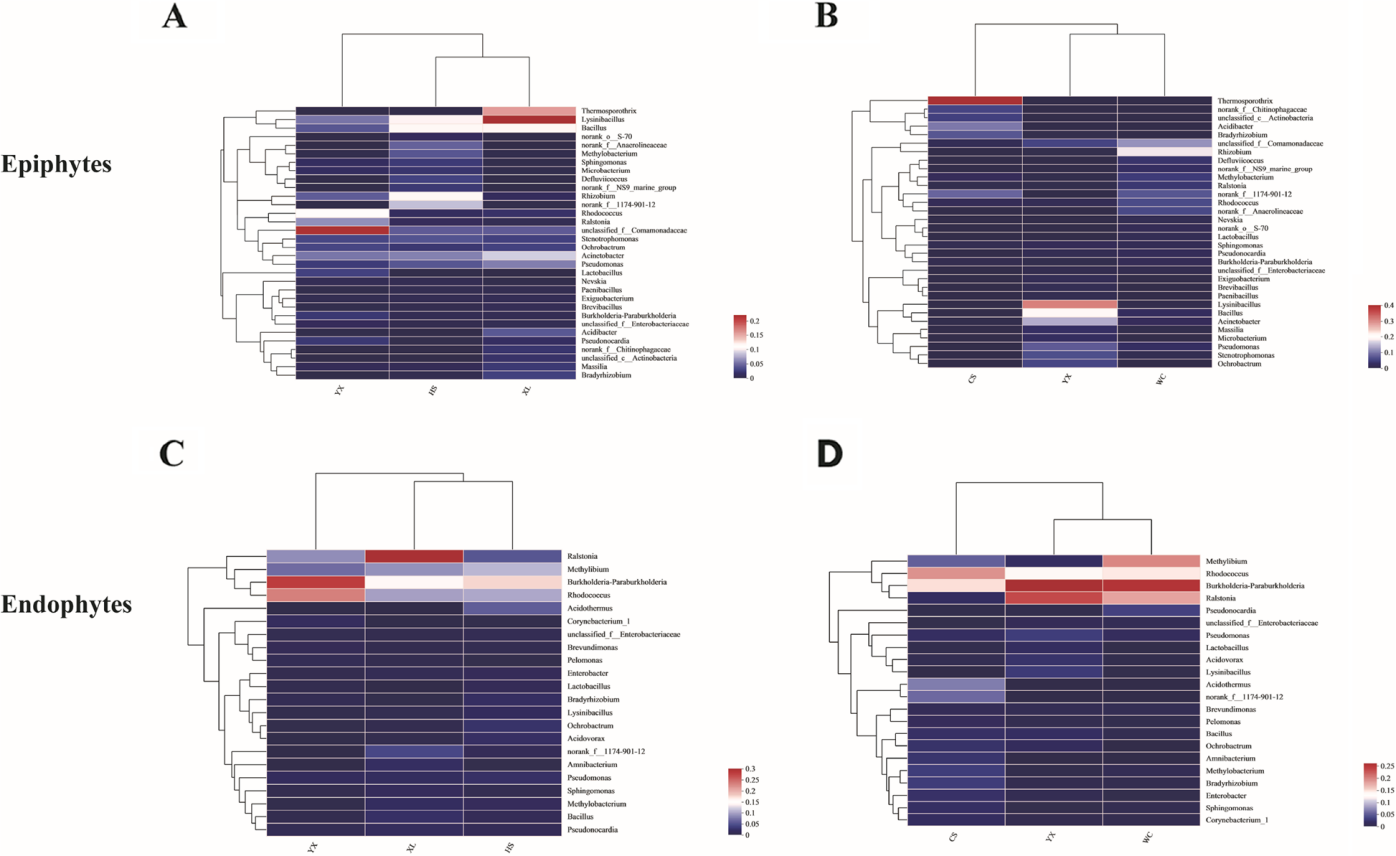
Heat map of bacterial community (genus) in epiphytic and endophytic bacterial of oil tea bacteria in different varieties and regions. XL: Xianglin210 HS: Huashuo YX: Youxian CS: Changsha WC: Wangcheng YX: Youxian. **A** the epiphytic community of the same regions with different cultivars, **B** the epiphytic community of the same cultivars in different regions, **C** the endophytes community of the same regions with different cultivars, **D** the endophytes community of the same cultivars in different regions

Cluster analysis and heatmap construction were performed on genera > 0.01% of bacterial communities in all samples (Fig. 3). For the epiphytic bacterial community, the community composition and relative abundance of different cultivars were notably different at the genus level. For example, for the ‘Xianglin 210’ samples, *Lysinibacillus, Thermosporothrix* and *Bacillus* were the dominant flora; for the ‘Huashuo’ samples, *Rhizobium*, *Bacillus* and *Lysinibacillus* were the dominant flora; for the ‘Youxian’ samples, Unclassfied_f_Comamonadaceae and *Rhodococcus* were dominant (Fig. 3A).

The epiphytic communities of samples planted in different areas showed great differences in community composition and relative abundance at the genus level. In terms of Changsha samples, *Thermosporothrix, Acidibacter* and *Bradyrhizobium* were dominant; in terms of Youxian samples, *Lysinibacillus, Bacillus, Acinetobacter* and *Pseudomonas* was dominant; in terms of Wangcheng samples, *Rhizobium*, unclassified_f__Comamonadaceae, *Rhodococcus* and norank_f__Anaerolineaceae were the dominant flora. In addition, *Thermosporothrix* (36.25%) had the largest relative abundance in Changsha samples; *Thermosporothrix* (28.13%), *Acidibacter* (12.51%) and *Acidibacter* (19.16%) accounted for the largest proportions in Youxian samples; *Rhizobium* accounted for the largest proportion in Wangcheng samples (Fig. 3B).

The leaf endophytic bacterial communities of different cultivars were basically similar in composition at the genus level, but different in relative abundance. For example, the relative abundance of *Ralstonia* was 29.54% in ‘Xianglin 210’, 8.42% in ‘Youxian’, 5.05% in ‘Huashuo’. The relative abundance of *Burkholderia* in ‘Youxian’ cultivar was 27.84%, which was clearly higher than that of ‘Xianglin 210’ (15.61%) and ‘Huashuo’ (17.47%). The relative abundance of *Rhodococcus* in ‘Huashuo’ (17.67%) was slightly higher than that in the other two cultivars (Fig. 3C).

For the endophytic bacterial communities of *C. oleifera* cultivars from different areas, the relative abundance of *Rhodococcus* was high (17.68%) in Changsha samples, while the relative abundance of *Ralstonia* was low (1.58%). The relative abundance of *Methylibium* was the highest in Wangcheng samples (18.32%). *Burkholderia* and *Ralstonia* had the highest relative abundances in Youxian samples (Fig. 3D).

**Fig.4:**
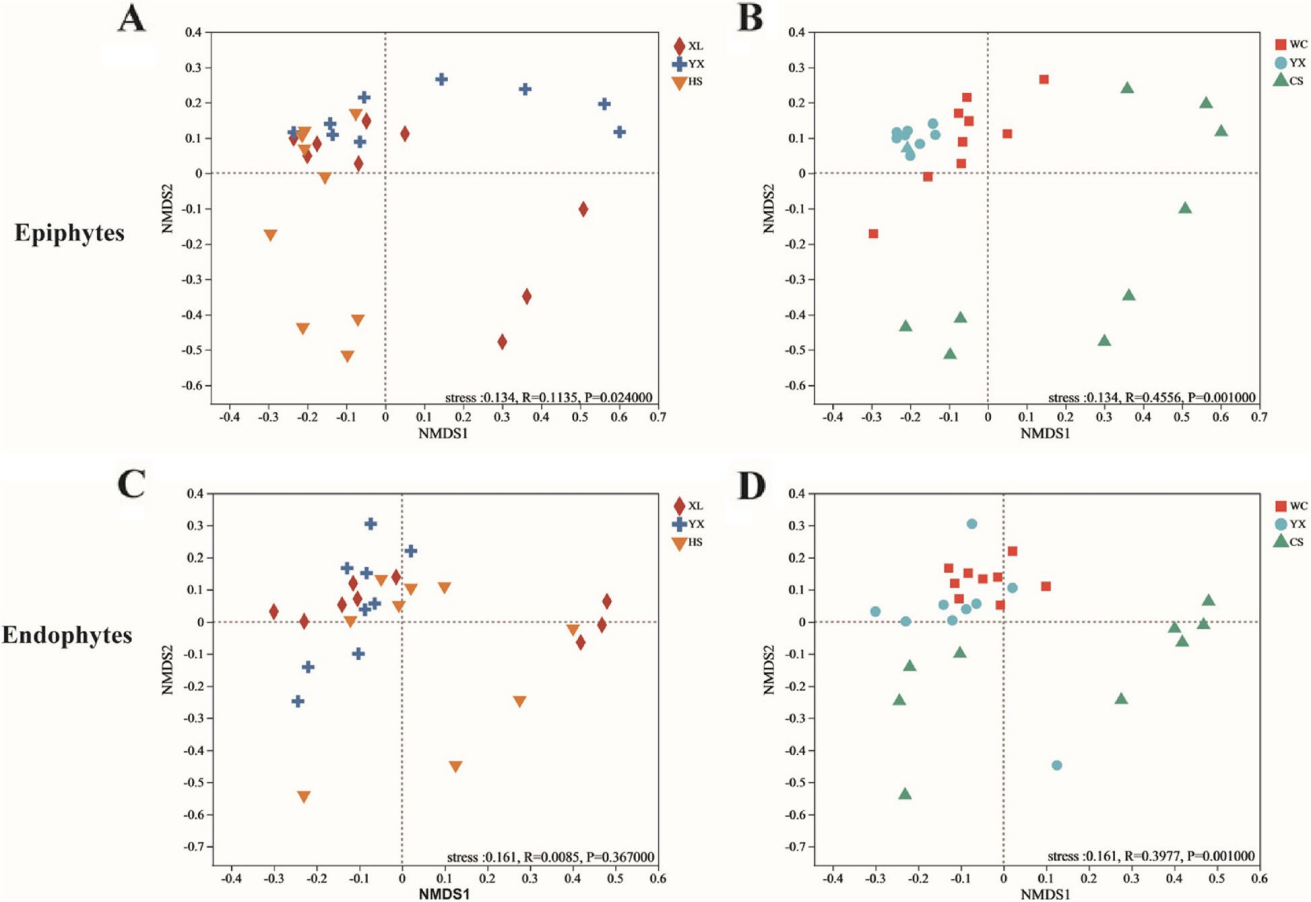
Non-metric multidimensional scale (NMDS) plots corresponding to the clustering of epiphytic and endophytic bacterial community. Cluster analysis was performed community similarity measures, namely, Jaccard coefficient (raw abundance data). XL: Xianglin210 HS: Huashuo YX: Youxian CS: Changsha WC: Wangcheng YX: Youxian. **A** the epiphytic community of the same regions with different cultivars, **B** the epiphytic community of the same cultivars in different regions, **C** the endophytes community of the same regions with different cultivars, **D** the endophytes community of the same cultivars in different regions

Differences in bacterial community structures were analyzed by non-metric multidimensional scaling (NMDS), and the distance between points represented the degree of bacterial community structure difference. As shown in Fig. 4, generally speaking, the distribution of both the epiphytic community and the leaf endophytic community were relatively concentrated, with small differences. The overall reduced dimensions are judged by the stress values. In this experiment, the stress values were 0.134 and 0.161, respectively, and the dimensionality reduction effect was good.

Analysis of similarity test (ANOSIM) was used to determine whether differences in bacterial community composition of samples were statistically significant. This analysis was performed from Jaccard (obtained from raw abundance data) with 999 permutations. ANOSIM generates an R-value ranging from 0 (completely similar) to 1 (completely dissimilar) and a *p*-value (significant level below 0.05) (Clarke, 2006). The difference of region (R = 0.4556, *p* = 0.001000) is greater than that of cultivars (R = 0.1135, *p* = 0.024000) in the bacterial community among epiphytic bacteria. Similarly, the region (R = 0.3977, *p* = 0.001000) has a greater influence on endophytic bacteria communities than on cultivars (R = 0.0085, *p* = 0.367000) (Fig. 4).

On the other hand, dissimilarity tests for each treatment using PERMANOVA methods based on Bray–Curtis distance, conducted to compare differences among region or plant cultivars under the epiphytes and endophyte community. There are significant differences among the bacterial communities in different regions among the epiphytes. Although there are significant differences between varieties and regions in endophytic bacterial communities of *C. oleifera*, the influence of regions on them is more significant (Table 3).

**Table 3:** Dissimilarity tests of epiphytic and endophytic bacteria communities using permutational multivariate analysis of variance (PERMANOVA) based on J Bray–Curtis distances

Part	Characteristics	Statistic (R^2^)	*p* Value
Epiphytes	Cultivar	0.10492	0.079
	Region	0.26632	0.001
Endophytes	Cultivar	0.19672	0.03
	Region	0.12013	0.001

Overall, although the external region and *C. oleifera* cultivars have effects on the epiphytic microbial and leaf endophyte communities, the richness and diversity of the epiphytes bacterial community are more affected by the external environmental factors. The composition of leaf endophytic bacterial community was more affected by the plant cultivars.

### Interaction network of epiphytes and endophytic microorganisms in *C. oleifera*

**Fig.5:**
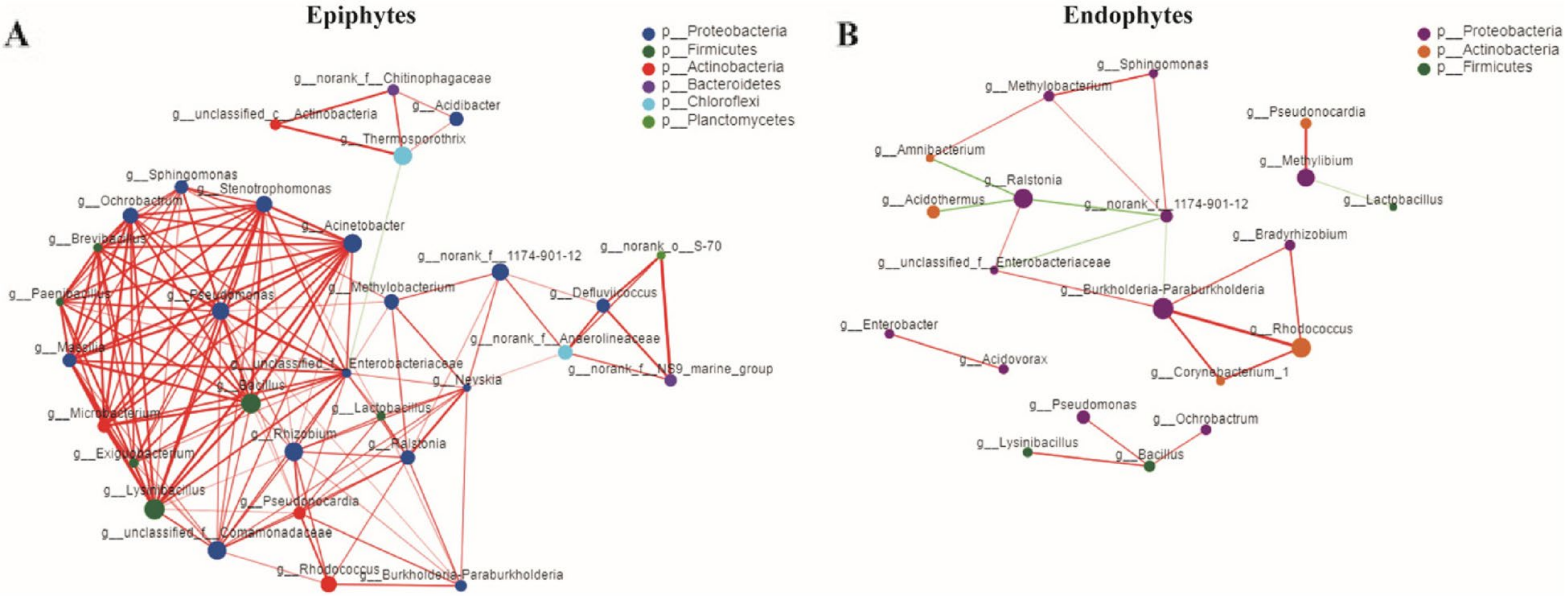
Microbial interaction networks in the different compartments. The interaction network of dominant microbiota at the genus level (≥ 0.01%) in epiphytic (**a**) and endophytic (**b**) bacterial community. The size of the nodes shows the abundance of OTUs, and the different colors indicate the corresponding taxonomic assignment at the phylum level. The edge color represents positive (red) and negative (green) correlations. The edge thickness indicates the correlation values; only significant interactions are shown (r > 0.6; *P* < 0.05)

Interactions between different microbiota are one of the main drivers of community structure and dynamics, as microbes can cooperate with each other or exclude each other. Networkx software was used to analyze the interaction network within the epiphytic and endophytic microorganisms of *C. oleifera*. At the genus level, Spearman correlation values between genera were calculated based on the occurrence patterns in the epiphytic and endophytic samples. The results showed that the nodal connectivity of the epiphytes microbial community was high. At the genus level (≥ 0.01%), there were 294 associations in epiphytic samples (Fig. 5A) and 44 associations in endophytic samples (Fig. 5B). According to the microbial interaction network constructed in this study, most of the microorganisms in the epiphytes bacterial community were positively interacting, which indicates that the symbiotic relationship was dominant in the epiphytic community, and the competitive relationship was weak. The leaf endophytic community was also dominated by symbiotic relationship, but the competition relationship was slightly enhanced. The proportion of symbiotic relationship in the epiphytes microbial community was 99.66%, and the proportion of competition relationship was 0.34%. The proportion of symbiotic relationship in leaf endophytic microorganisms was 88.64%, and the proportion of competition relationship was 11.36%. Overall, in the two microbial networks, the epiphytic microbial network was the most closely connected and had the most complex structure. It can also be seen from Fig. 5 that there were differences in the size of each module, and larger node modules were formed in the epiphytic microbial network to maintain the structure and function of the network.

In addition, according to the network topology analysis, the top 5 microorganisms within centrality of the epiphytic microbial interaction network, that had been identified included *Exiguobacterium*, *Methylobacterium*, *Paenibacillus*, *Pseudonocardia* and *Ochrobactrum*, and as for the leaf endophytic microbial interaction network, it included *Acidovorax, Enterobacter*, *Amnibacterium*, *Methylobacterium* and *Burkholderia*-*Paraburkholderia*. These microbes were highly central and related, and might be the key microorganisms to maintain the stability of the microbial ecological network.

### Function prediction of epiphytes and leaf endophytic microorganisms in *C. oleifera*

**Fig. 6:**
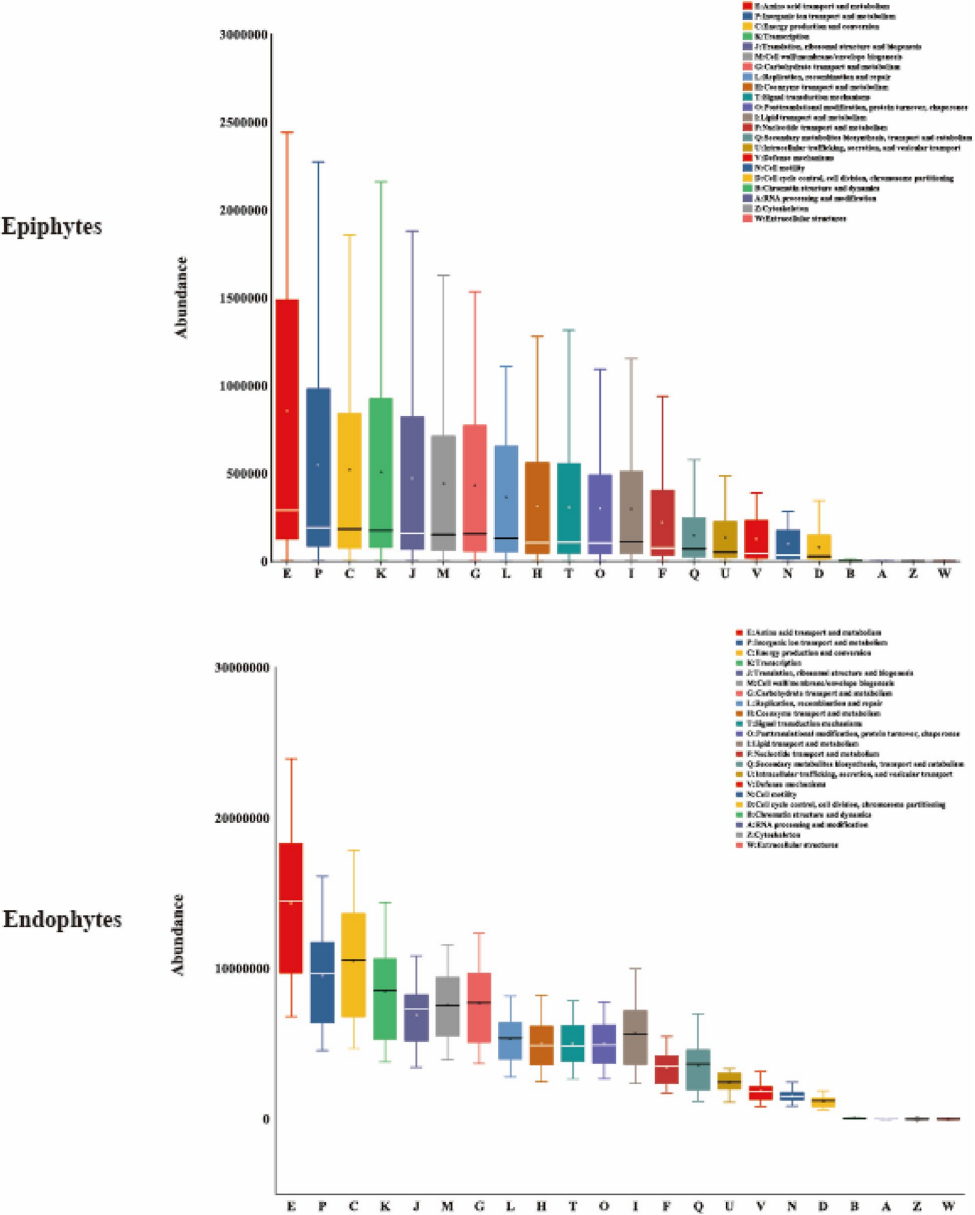
Functional prediction of COG

**Fig. 7:**
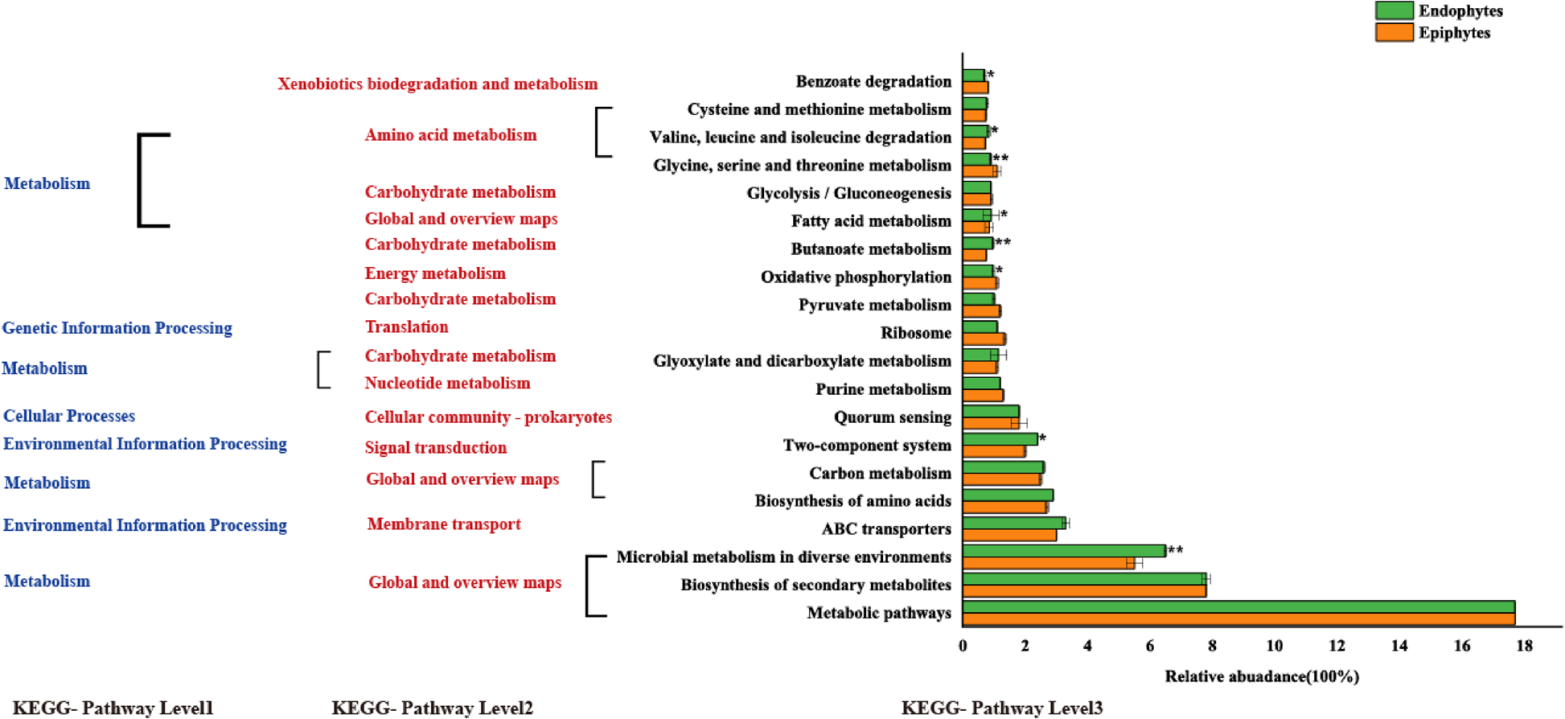
Functional predictions for epiphytic and endophytic bacterial community with significantly different KEGG pathways (*P* < 0.05). KEGG pathways at Level 1, Level 2, and Level 3 are represented. *0.01 < *P* < 0.05, **0.001 < *P* < 0.01

All epiphytes and endophytic bacterial communities had similar COG function classification patterns as generated by PICRUSt. There were higher relative abundance sequences related to amino acid transport and metabolism, energy production and conversion, transcription (Fig. 6). Prediction software PICRUSt2 enriched 13 categorizable dominant pathways (relative abundance > 1%) in the KEGG pathway level 3. The relative abundance of metabolic pathways (17.7%) was the highest in endophytes and epiphytes, followed by biosynthesis of secondary metabolites (7.8%), microbial metabolism in diverse environments (5.5%, 6.5%), and ABC transporters (3%, 3.3%) (Supplementary Tab 1). Eight pathways had significant differences between endophytes and epiphytes (*P* < 0.05). It is worth noting that there were significant differences in the relative abundance of the microbial metabolism in diverse environments, the butanoate metabolism and glycine, serine and threonine metabolism (Fig. 7).

## Discussion

The epiphytic is an ecological environment rich in microorganisms. The epiphytes microorganisms together with their living environment constitute a complex ecosystem, which plays an extremely important role in the growth and development of host plants. Studies have shown that the community structure and diversity of plant epiphytes microorganisms are affected by plant species, host genotypes, seasons, geographic locations, and environmental factors [33, 34]. Likewise, endophytes are widely present in various tissues and organs of plants and are an important part of the plant micro-ecosystem, which plays an essential role in the growth and health of host plants. The community diversity of endophytes depends not only on host plant genotype and endophyte species, but also on geographic location, climatic conditions, soil type, nutrient stress, temperature, rain, air humidity, etc. [35]. In this study, based on high-throughput sequencing technology, the epiphytic and endophytic bacterial diversity and community structure characteristics of three *C. oleifera* cultivars from three different areas were analyzed. In the same region, the diversity and richness of epiphytic and leaf endophytic bacterial communities of different *C. oleifera* cultivars were basically the same. Moreover, the diversity and richness of leaf endophyte community were significantly higher than those in epiphytic community.

There were significant differences in the diversity and composition of the epiphytes bacterial community among different *C. oleifera* cultivars. Due to the closer interaction between endophytic bacteria and host plants, the leaf endophytic bacterial community is more significantly affected by the variety, and the epiphytic microbial community is more affected by environmental factors. For example, Wang et al. [36] studied the community structure and diversity of endophytic bacteria in 10 rice cultivars, and the results showed that the genotype of rice seeds had a greater impact on the abundance and diversity of endophytic bacteria. Zhiming [37] study on characteristics of main meteorological and soil factors and the relationship with the style of flue-cured tobacco in baoshan. He made a preliminary analysis on the endophytic bacterial communities of tobacco of different cultivars and different growth periods, and proposed that the endophytic bacterial communities in different tobacco cultivars were different, and the endophytic bacterial communities changed with the different stages of plant growth and development.

The planting geographic locations and cultivars of *C. oleifera* had a critical impact on the bacterial community structures. Differences in nutritional and environmental conditions within or on the surface of *C. oleifera* leaves resulted in significant differences in the composition of epiphytic and leaf endophytic bacterial communities. In this study, compared with the leaf endophytic community, a large number of *Actinomycetes* and *Firmicutes* were found in the epiphytic bacterial community. Compared with the living environment of endophytic bacteria, the epiphytic is exposed to external conditions such as ultraviolet light and drought, so the bacterial members resistant to desiccation and radiation are predominant. Many previous studies showed that when *Actinobacteria* and *Firmicutes* dominated the bacterial community, the hosts were more resistant to UV light and desiccation [38]. This resistance was mainly attributed to the production of photoprotective pigments and repair of UV damage through multiple mechanisms [39]. Furthermore, their ability to produce spores allowed them to survive harsh conditions [40]. The results of this study showed that, compared with the epiphytic bacterial communities of the other two areas, *Actinomycetes* and *Firmicutes* had greater advantages in the epiphytic bacterial communities. Previous studies have also proved that *C. oleifera* in Youxian area had strong disease resistance [41], which was consistent with the results of this study.

The research on the mutualistic relationship between the *C. oleifera* microbial community and the host plants mainly focuses on the rhizosphere microbial community, while the epiphytic microbial community is rarely studied. Song [41] analyzed the rhizosphere microbial community structure of *C. oleifera* ‘Youxian’ and found that the rhizosphere soil bacteria of *C. oleifera* ‘Youxian’ mainly included *Chloroflexi*, *Proteobacteria*, *Acidobacteria*, *Actinobacteria*, *Planctomycetes* and *Firmicutes*. In this study, we analyzed the community structure of *C. oleifera* cultivars, it was concluded that the dominant flora in the epiphytic mainly included *Proteobacteria*, *Actinobacteriota*, *Firmicutes* and *Chloroflexi*. The endophytic dominant flora of *C. oleifera* cultivars mainly included *Proteobacteria*, *Actinobacteria* and *Firmicutes*, which was similar to the epiphytic bacterial community structure in previous studies. For example, Müller et al. [42] found a large number of microorganisms belong to *Proteobacteria*, *Actinobacteria* and *Firmicutes* on the surface of olive leaves. Valverde et al. [43] also found that the dominant flora in chestnut belong to *Proteobacteria*, *Actinobacteria* and *Firmicutes*. The main difference in the composition of the dominant flora of the two communities was *Chloroflexi*. Many studies have shown that *Chloroflexi* is beneficial to the fixation of CO_2_ in plants, and some studies have shown that the photoautotrophic *Chloroflexi* has a close interaction with other microbes in the community [44–46]. *Chloroflex* abundance was higher in the epiphytic community because photosynthesis of leaves was mainly carried out on the leaf surface.

During the long-term co-evolution process, the epiphytic microbes and leaf endophytes have formed a mutualistic relationship with the host plants. The plants can provide nutrients for the growth of microorganisms, and the microorganisms can help the host resist the adverse environment by synthesizing secondary metabolite [47], and promoting plant growth. Interactions between biological communities play a key role in maintaining the function and stability of ecosystems. In this study, based on the Networkx software, we analyzed and constructed the interaction network between the epiphytic and the endophytic microbial communities of *C. oleifera*, and revealed the characteristics of the epiphytic and the endophytic microbial communities. Through Networkx software analysis have shown that the epiphytes microbial network is larger and more complex. Studies have shown that the phyllosphere microbial network is larger and more complex [48], but its response speed is faster and it is easily disturbed by the external environment factors. According to Fig. 5, most microorganisms have positive interactions. This indicates that the symbiotic relationship is dominant in the phyllosphere microbes, and the competitive relationship is weak. In addition, this study screened the microbes with the highest centrality value and played a key role in the microbial communities, such as *Exiguobacterium*, *Methylobacterium*, *Paenibacillus*, *Pseudonocardia* and *Ochrobactrum* for the phyllosphere community, and *Acidovorax*, *Enterobacter*, *Amnibacterium*, *Methylobacterium* and *Burkholderia*-*Paraburkholderia* for the leaf endophytic microbial community. Among them, *Paenibacillus* [49] is an important source of plant growth-promoting bacteria, which can directly promote plant growth through mechanisms such as nitrogen fixation, hormone production, siderophore secretion, and activation of mineral nutrients, and other mechanisms to defense against plant diseases. *Burkholderia* [50] can colonize the plant surface and rhizosphere, fix nitrogen, dissolve phosphorus, reduce plant ethylene levels, and produce auxin. *Methylobacterium* [51] is a G-bacteria with both methylotrophic and methanotrophic properties. It plays an important role in the natural carbon cycle and effectively degrades organic phosphorus, methamidophos and other substances in the soil. *Enterobacter* [52] can promote the growth of some plants, and at the same time, it can be used as a biological pesticide, which is important in green biological control. It shows that there are abundant probiotics in the epiphytic and leaf endophytic microbial communities of *C. oleifera*.

The functional structure of microbial communities is intricately linked to the environmental factors present in their respective habitats [53–55]. In this study, we used PICRUSt2 to predict the function of epiphytic and endophytic microbial communities of *C. oleifera*. Our results have shown that the top three in relative abundance and the top three in significant differences of the KEGG-pathway level 3 belong to metabolism in the KEGG-pathway level 1. The relative abundance of two-component system and ABC transporters pathways for endophytes were higher than epiphytes. This pathways belongs to environmental information processing. This suggests that endophytic bacteria may have a stronger relationship with the environment. This environment mainly refers to the plant species, health status, growth stage, etc. Therefore, this can also reflect a closer relationship between endophytic bacteria and cultivars.

## Conclusion

In this study, the epiphytes and endophytic microbial community of *C. oleifera* was mainly affected by environmental factors. The diversity and richness of leaf endophytic community was significantly higher than that of epiphytes microbial community. The epiphytes microbial network is the most closely connected and the most complex in structure.

### Supplementary Information

Supplementary Material 1.

## Data Availability

The datasets generated during and analysed during the current study are available from the corresponding author on reasonable request. The datasets generated and/or analysed during the current study are available in the NCBI repository, (Accession Number: SRA: PRJNA948360). http://www.ncbi.nlm.nih.gov/bioproject/948360
