# Supplementary figures and images for: Epiphytic and endophytic bacteria on Camellia oleifera phyllosphere: exploring region and cultivar effect
Source: BMC Ecol Evol. 2024 May 13;24:62. doi: 10.1186/s12862-024-02240-3 (PMC11089727; doi:10.1186/s12862-024-02240-3)

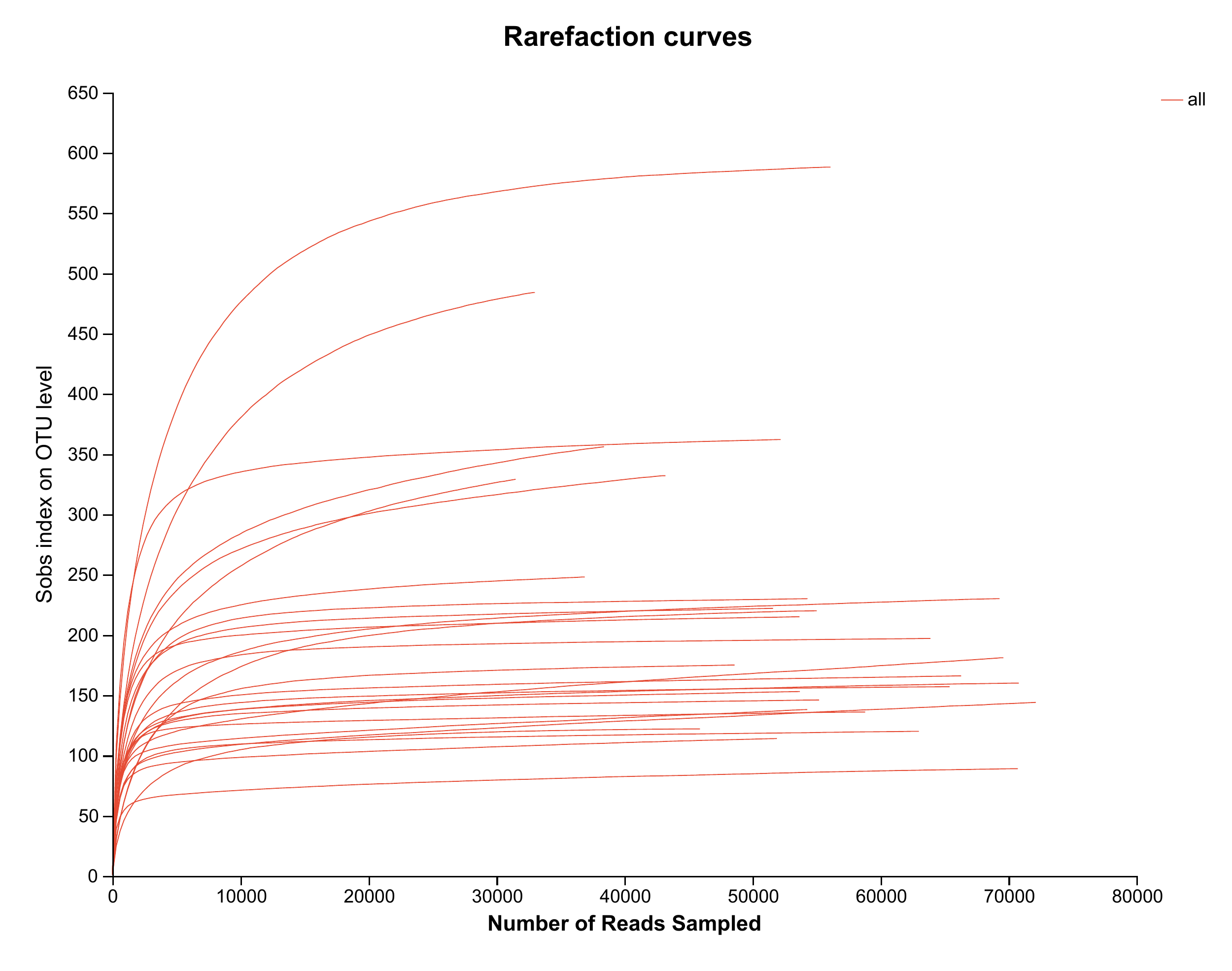

Supplement: Supplementary file 1 — Supplementary Material 1. [file 12862_2024_2240_MOESM1_ESM.zip › Rarefaction curve.tiff]
