# Supplementary material for: Epiphytic and endophytic bacteria on Camellia oleifera phyllosphere: exploring region and cultivar effect
Source: BMC Ecol Evol. 2024 May 13;24:62. doi: 10.1186/s12862-024-02240-3 (PMC11089727; doi:10.1186/s12862-024-02240-3)
Supplement: Supplementary file 1 — Supplementary Material 1. [file Author mail.pdf › Author mail.pdf]

Adrielene Cutamora

**From:**	何苑皞 <heyuanhao218@163.com>
**Sent:**	Saturday, May 4, 2024 6:06 PM
**To:**	Adrielene Cutamora
**Subject:**	Re:RE: BMC Ecology and Evolution: 12862_2024_2240 - Missing supplementary materials
**Attachments:**	kegg.xlsx; Rarefaction curve.tiff

This Message Is From an External Sender
This message came from outside your organization.
Report Suspicious

Dear Adrielene,

Hope all is well.
My apologies for the late reply. I'm very sorry for missing the supplementary. Now 'Supplementary Fig. 1 and Supplementary Table 1' were attached to the e-mail.

I am very pleased to hear from you.

Best wishes!

Sincerely

Yuanhao He

At 2024-05-03 13:16:56, "Adrielene Cutamora" <adrielene.cutamora@springernature.com> wrote:

> Dear Dr. Yuanhao He,
>
> Hope all is well.
>
> We would like to ask if you happen to receive the email below.
>
> Thank you and we look forward to hearing from you.
>
> Best regards,
> Adrielene
>
> ---
> **Adrielene G. Cutamora**

Production Supervisor
https://www.straive.com
LP Hypermart IT Park | Dumaguete City | Negros Oriental | Philippines 6200
adrielene.cutamora@springernature.com

Straive is a service provider of **Springer Nature**

From: Adrielene Cutamora
Sent: Tuesday, April 30, 2024 2:47 PM
To: heyuanhao218@163.com
Cc: Ma, Amy <amy.ma@cn.biomedcentral.com>
Subject: BMC Ecology and Evolution: 12862_2024_2240 - Missing supplementary materials

Dear Dr. Yuanhao He,

We are reaching out to you with reference to your article, which will soon be published in *BMC Ecology and Evolution*. However, before we proceed, we would like to inform that 'Supplementary Fig. 1 and Supplementary Table 1' were mentioned in the manuscript; however, e-files were missing in the source package. Kindly advise on how we may proceed.

Please feel free to reach out to us if you have any additional questions.

Thank you.

Title: Epiphytic and Endophytic Bacteria on Camellia oleifera Phyllosphere: Exploring Region and Cultivar Effect
DOI: 10.1186/s12862-024-02240-3

Best regards,
Adrielene

Adrielene G. Cutamora
Production Supervisor
https://www.straive.com
LP Hypermart IT Park | Dumaguete City | Negros Oriental | Philippines 6200
adrielene.cutamora@springernature.com

Straive is a service provider of **Springer Nature**
